# Building professionals’ intention to use smart and sustainable building technologies – An empirical study

**DOI:** 10.1371/journal.pone.0201625

**Published:** 2018-08-01

**Authors:** Wai-Ming To, Peter K. C. Lee, King-Hang Lam

**Affiliations:** 1 School of Business, Macao Polytechnic Institute, Macao SAR, China; 2 Department of Logistics and Maritime Studies, The Hong Kong Polytechnic University, Hong Kong SAR, China; 3 Department of Electrical and Electronic Engineering, The University of Hong Kong, Hong Kong SAR, China; Universitas Indonesia, INDONESIA

## Abstract

Smart and sustainable buildings save energy and material resources and provide a comfortable environment that enhances their occupants’ well-being and productivity. It is therefore crucial to understand how building professionals, including designers, engineers, and contractors, view smart and sustainable buildings and what drives them towards smart and sustainable building technologies. This study identifies salient smart and sustainable building features from building professionals’ perspective and explores what determines building professionals’ intention to use such building technologies. Responses from 543 Hong Kong’s building professionals identify that intelligent security, intelligent and responsive fresh air supply, and intelligent and responsive thermal control are among the most important features of smart and sustainable buildings. Results of structural equation modeling grounded on an extended technology acceptance model indicate that facilitating condition and job relevance are related to perceived ease of use while subjective norm pertaining to image and perceived ease of use are predictors of perceived usefulness. Facilitating condition, perceived ease of use and perceived usefulness jointly influence building professionals’ intention to use smart and sustainable building technologies.

## Introduction

Owing to improvements in their physical design and use of modern information and communication technology (ICT) installations, smart and sustainable buildings are becoming more intelligent, responsive, and adaptive to the changing needs of building users over their life cycles. By being adaptive, they optimize the use of energy, water, and material resources and provide comfortable and responsive environments to their occupants [1–3]. Smart and sustainable buildings can also serve as active components of smart grid that consume, store and produce energy [4]. Siemens [2] indicated that the use of smart and sustainable building technologies could reduce the energy consumption of buildings by 30 percent. Since Hong Kong’s commercial electricity consumption is currently about 30 billion kWh/year and 90 percent of it is consumed in commercial buildings [5] and the emission factor of Hong Kong’s electricity is 0.722 kg CO_2_-eq/kWh, it should be possible to reduce Hong Kong’s greenhouse gas emissions by over 5 million tons (MT) of CO_2_-eq/year [6]. However, energy saving is only one of the many features of smart and sustainable buildings. Smart and sustainable buildings are equipped with smart metering, information and communications systems, security systems, and intelligent and responsive systems that can interact with building occupants and ambient conditions to provide a comfort indoor environment in different seasons [1,7].

Although smart and sustainable buildings have numerous advantages, such as increasing building value and saving energy, water, material resources and money, smart and sustainable buildings and their features cannot be pinned down easily because technologies are evolving constantly [1,8] and human factors must be catered for [9,10]. According to Buckman et al. [1], progress in smart and sustainable buildings is being driven by four drivers: intelligence (level and degree of intelligence), enterprise (firms using the buildings), materials and design (the physical forms of buildings), and control (interactions between occupants and buildings). Overall, the core element of smart and sustainable buildings is ‘adaptability’. Hoy [10] indicated that smart and sustainable building technologies should enable the optimal use of lighting, heating, and cooling, provide a secure and comfortable environment, improve the mobility of occupants, and accommodate changes in network infrastructure. Jang et al. [11] indicated that the building facade has played a significant role in shaping indoor environmental conditions such as natural lighting, thermal control, natural ventilation, and noise control. They suggested that intelligent and responsive facade and other sensing and actuating elements shall be deployed to enhance the adaptability of smart and sustainable buildings. Arditi et al. [12] examined the smartness of buildings from the perspectives of building owners, designers, and contractors. They established a smartness index that focused on three groups of attributes, namely, economic issues arising from building design, construction, and operational perspective; energy issues arising from heating, cooling, lighting, and plumbing systems; and comfort issues concerning air quality, temperature, humidity, acoustic, functionality, security, and fire protection. Arditi et al. [12] reported that building owners, designers, and contractors emphasized most on energy issues, followed by economic issues and then comfort issues. Besides, the social and ecological functions of smart and sustainable buildings cannot be ignored because many people spend most of their waking hours in workplaces [13]. Yet, we are still in the dark about the relative importance of different features of smart and sustainable building. The first objective of the study is to identify the salient features of smart and sustainable buildings from the perspectives of building professionals. Also, the building and construction industry is known for its reluctance to change, particularly for adopting new technologies [14]. Thus, the second objective of the study is to apply the extended Technology Acceptance Model (TAM)–one of the most popular theoretical models predicting the adoption of technologies [15,16]–to explore whether building professionals’ perceptions of smart and sustainable building technologies such as subjective norm, image, job relevance influence their perceived ease of use, perceived usefulness, and behavioral intention to use smart and sustainable building technologies. According to Davis et al. [17], an individual’s intention to adoption a new technology depends significantly on his/her perceptions of the ease of use and the usefulness of that technology. The antecedents of users’ perceived ease of use and perceived usefulness may include subjective norm, image, job relevance, and facilitating conditions [15,16]. The results of the study shed new insights on what factors drive building professionals to use smart and sustainable building technologies.

The rest of the paper is organized as follows. The next section presents a brief historical review of the development of smart and sustainable buildings, the extended TAM, and the formulation of hypotheses. The section to follow describes the method including the survey questionnaire, data collection procedure, and analysis approach, followed by the results and discussion. The paper ends with conclusions and certain suggestions for future work.

## Literature review and hypothesis development

### Smart and sustainable buildings

The predecessors of smart and sustainable buildings, i.e., intelligent buildings have been researched widely over the past three decades [1,18–21]. Building professionals such as designers, engineers, and academics of the 1990s had focused primarily on the development and deployment of automated building systems that improve technical performance, minimize cost, and enhance flexibility [22]. Intelligent Building Institute has defined intelligent buildings as the ones that integrate different building systems to manage resources in a coordinated manner to enhance technical performance, flexibility, capital and operating cost savings from building owners’ and managers’ perspectives [18,23]. Most of these features were centered on technological aspects but building occupants’ needs, wants, and control were almost ignored. Nevertheless, following technological aspects, in particular, the emergence of ICT, building professionals realized that intelligent buildings must respond to occupants’ needs and requirements [18,19,24]. Researchers suggested that intelligent buildings should be sustainable, healthy, flexible, and adaptable to the changing needs of occupants (be they people or organizations) [19,25]. With the growing use of smart devices, smart and sustainable building systems, and cloud computing, new intelligent buildings have become more responsive to the needs of occupants and changing environmental conditions. This is why these buildings started being referred to as smart and sustainable buildings [1,21]. Smart and sustainable building technologies encompass a wide range of evolving smart devices, systems, and platforms. Siemens [3] stated that smart and sustainable buildings would increase energy efficiency, cost efficiency, grid reliability, and building value while ensuring long-term sustainability. Smart and sustainable building technologies are most about how to use the collected data from smart components to facilitate occupants to conduct their activities effectively in a safe, comfortable, and healthy environment. They include intelligent building automation systems, responsive facades and roofs, and configurable indoor green and social spaces [1,3,11,12]. Arditi et al. [12] conducted a questionnaire survey to solicit opinions from members of the US Construction Management Association and suggested that smart and sustainable building features can be grouped as those dealing with energy, economic, and occupant comfort issues. They then developed a smartness index of buildings from the perspectives of building designers and owners. In addition, intelligent and responsive building envelope, and the social and ecological elements of building design will have significant impact on building users’ physiological and psychological comfort [11,13]. The present study solicits and analyzes Hong Kong building professionals’ opinions on the relative importance of smart and sustainable building features.

### The extended technology acceptance model

TAM is the most popular theory explaining user acceptance and behavior related to computer and ICT technologies [26–28]. TAM and its extended versions have been applied to health care [29,30] and knowledge transfer in the mobile environment [31]. Davis [32] developed TAM by identifying the key antecedents of user acceptance of a new technology. According to TAM, users evaluate the new technology based on perceived ease of use and perceived usefulness. Perceived ease of use refers to the degree to which using the technology is free of effort. Perceived usefulness refers to the extent to which using the technology enhances task performance [17,32]. When a technology is perceived to be easy to use and useful, users will have positive attitude towards using the technology and high behavioral intention to use the technology [17]. Finally, the adoption of technology is determined by users’ behavioral intentions. It should be noted that, to keep it parsimonious, the final model of TAM excludes the attitude construct [17,33]. Accordingly, it is posited that:

H1: Perceived ease of use positively influences perceived usefulness in using smart and sustainable building technologies.
H2: Perceived ease of use positively influences behavioral intention to use smart and sustainable building technologies.
H3: Perceived usefulness positively influences behavioral intention.

The main advantage of TAM was (and still is) its simplicity. Subsequently, some researchers extended it by including other constructs such as subjective norm, perceived behavioral control, facilitating condition, and self-efficacy [34–36]. Venkatesh and Davis [15] extended TAM by adding social influences such as subjective norm and image, and cognitive processes such as job relevance, result demonstrability, and output quality as antecedents of perceived usefulness. Perceived ease of use is influenced by users’ beliefs including self-efficacy and facilitating condition of the external environment [16,37]. Self-efficacy refers to users’ perceived capabilities to perform a specific task. Facilitating condition refers to the degree to which users believe that organizations provide appropriate infrastructures to facilitate the use of the new technology. The present study however includes only the facilitating condition because the use of smart and sustainable building technologies depends significantly on how well organizations support building professionals to use new technology. Indeed, facilitating condition influences professionals’ perceived ease of use and behavioral intention. We do not include self-efficacy because most building professionals have sufficient understanding on smart and sustainable building technologies. Hence, the following hypotheses are posited:

H4: Facilitating condition positively influences perceived ease of use.
H5: Facilitating condition positively influences behavioral intention.

Venkatesh and Davis [15] suggested that the antecedents of perceived usefulness also include subjective norm, image, job relevance, result demonstrability, and output quality. Since the use of smart and sustainable building technologies is still relatively new on the learning curve, result demonstrability and output quality that require many years of data to prove them were excluded. Subjective norm refers to users’ perceptions that people who are important to them think they should use the new technology. Image refers to the degree to which the use of the new technology is perceived to enhance users’ status and image in the social system [38]. Job relevance refers to the extent to which the new technology is applicable to users’ jobs. Since users are often influenced by subjective norms to establish a favorable image about the technology, known as the identification process as put forward by Kelman [39], Venkatesh and Davis [15] suggested that subjective norm is not only an antecedent of perceived usefulness; it also influences image. Thus, based on the above studies, it is posited that:

H6: Subjective norm positively influences perceived usefulness.
H7: Subjective norm positively influences image.
H8: Image positively influences perceived usefulness.
H9: Job relevance positively influences perceived usefulness.

**Fig 1:**
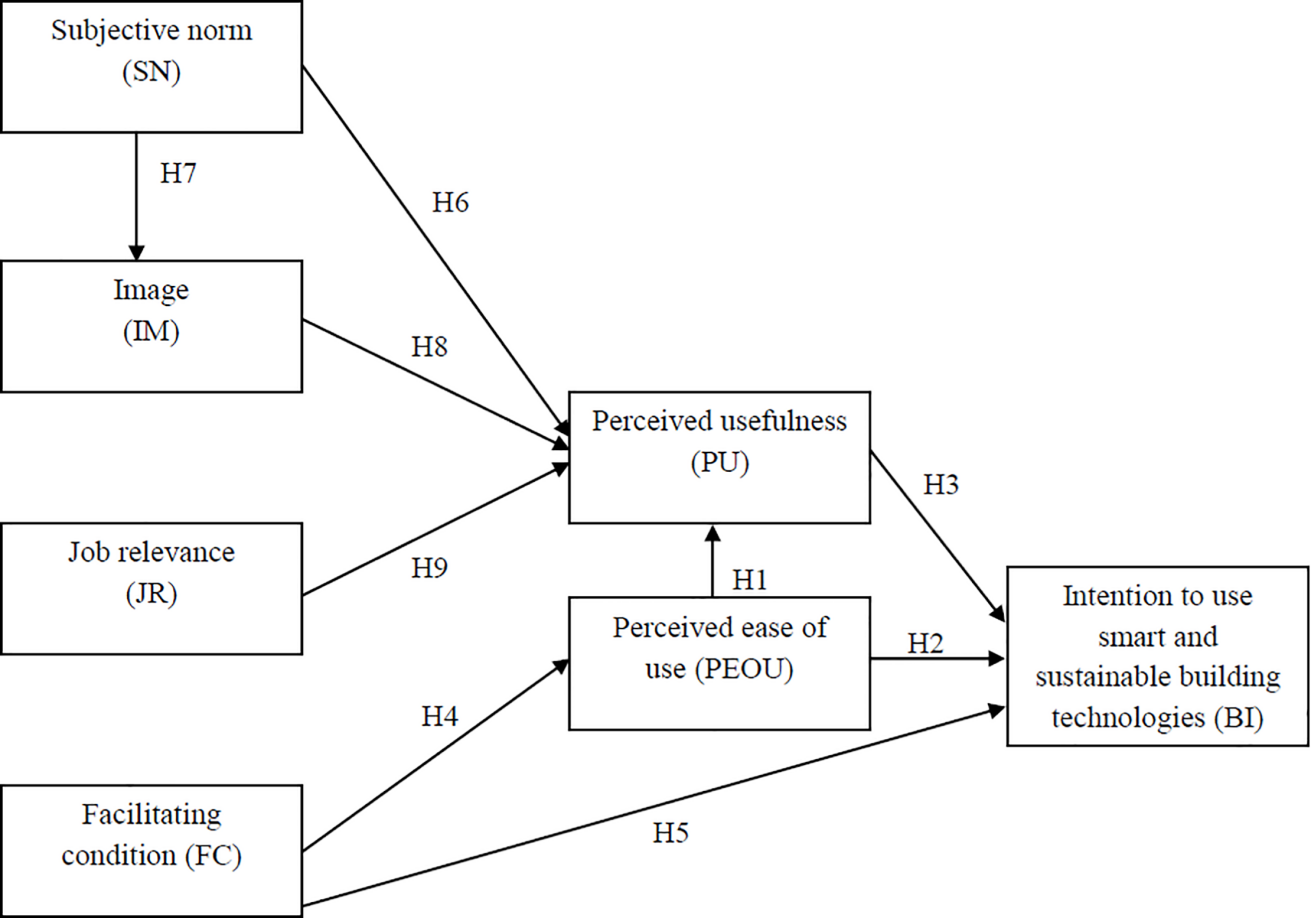
The theoretical model of the study.

Fig 1 shows the theoretical model of the study. All hypotheses are also shown in Fig 1.

## Method

A cross-sectional survey was used to obtain inputs from building professionals. A structured self-administered questionnaire was developed and the questionnaire was used to collect data from employees of Hong Kong’s engineering companies, construction companies, building management companies, and building owners.

### Questionnaire design

The questionnaire had three sections. The first section included the 17 smart and sustainable building features shown in Table 1. Question items were obtained from the extant literature [1,11–13,18,19]. The items cover intelligent and responsive building systems such as fresh air supply, thermal control, lighting, expandable information and communications network, security system, etc. [1,12,18,19]; responsive elements to deal with changes in the building’s external environment such as smart power grid, daylight, rain, etc. [11,18,19]; and ecological and social features of the building [13]. Each feature was rated using a 5-point Likert scale, where ‘1’ represents ‘not at all important’ and ‘5’ represents ‘very important’. In the second section, respondents were asked to consider the smart and sustainable features listed in the first section as a group of smart and sustainable building technologies. This section consisted of 22 items measuring seven constructs: Facilitating Condition (FC), Subjective Norm (SN), Image (IM), Job Relevance (JR), Perceived Ease of Use (PEOU), Perceived Usefulness (PU), and Behavioral Intention (BI). FC that characterizes users’ perception of the support from the organizations to adopt the new technology was operationalized using three items adapted from Gu et al. [16]. SN measures the influence from other people on users’ adoption of the technology, IM measures users’ perceived impact of the adoption of the technology on the enhancement of their image and status, and JR measures whether the technology is applicable to their job. They were operationalized using nine items (three items for each construct) from Venkatesh and Davis [15]. PEOU characterizes users’ perception of the ease of use, PU characterizes users’ perception of the usefulness of the new technology, and BI determines users’ intention to use the technology. They were operationalized using four items, three items, and three items respectively from Davis [32], Davis et al. [17], and Venkatesh and Davis [15]. The 22 items are shown in Table 2. Each of these items was rated using a 5-point Likert scale, where ‘1’ represents ‘strongly disagree’ and ‘5’ represents ‘strongly agree’. The third section consisted of five questions that sought the demographic characteristics of respondents including gender, age group, education, job position, and work experience in the organizations.

**Table 1:** Smart and sustainable building features.

Items	Mean	SD
An intelligent security system is … Intelligent and responsive fresh air supply is … Intelligent and responsive thermal control is … Intelligent and responsive lighting is … Intelligent transport systems such as lifts and escalators are … Having minimum impacts on the environment is … An expandable network infrastructure is … A responsive facade that can be used to harvest daylight is … A building information system is … An intelligent system that can respond to smart power grid is … An indoor social space (to balance work and life) is … An intelligent energy system that can harvest solar and wind … Different social venues that facilitate user interaction are … A ‘real’ indoor green space (with a variety of plants) is … Responsive acoustic environment is … A responsive system that can be used to harvest rainwater is … An intelligent system that monitors people movement is …	4.18 4.03 4.02 3.99 3.94 3.88 3.83 3.75 3.74 3.71 3.71 3.69 3.64 3.59 3.57 3.54 3.36	0.92 0.94 0.89 0.86 0.99 0.97 0.93 0.98 0.93 0.90 0.99 1.06 0.84 0.95 0.88 0.99 1.02

Note: Each item was rated using a 5-point Likert scale in which ‘1’ represents ‘not at all important’ and ‘5’ represents ‘very important’.

**Table 2:** Measurement items, means and SD of items, and factor loadings from CFA.

Code	Item	Mean (SD)	Factor loadings
	*Facilitating condition*		
FC1	I have the knowledge necessary to adopt/design/use smart and sustainable building tech.	3.36 (0.96)	0.70
FC2	I have the resources necessary to build and provide smart and sustainable building tech.	3.15 (1.01)	0.80
FC3	A specific group is available to assist with smart and sustainable building tech’s appl.	3.28 (0.97)	0.76
	*Subjective norm*		
SN1	My seniors think I should use smart and sustainable building tech.	3.29 (0.87)	0.74
SN2	My colleagues think I should use smart and sustainable building tech.	3.24 (0.87)	0.80
SN3	My professional friends think I should use smart and sustainable building tech.	3.49 (0.92)	0.68
	*Image*		
I1	People using smart and sustainable building tech. have more prestige than those who do not.	3.46 (0.95)	0.73
I2	People using smart and sustainable building tech. have a high profile.	3.45 (0.96)	0.75
I3	Adopting/designing smart and sustainable is a status symbol.	3.51 (0.94)	0.69
	*Job relevance*		
JR1	Use of smart & sustainable build. tech. is relevant to building design and management.	3.95 (0.85)	0.75
JR2	Use of smart & sustainable build. tech. is important to building design and management.	3.84 (0.82)	0.83
JR3	Use of smart & sustainable build. tech. is useful to building design and management.	3.82 (0.85)	0.70
	*Perceived ease of use*		
PEOU1	The interaction between people and smart & sustain. build. would be clear & understandable.	3.69 (0.83)	0.66
PEOU2	Smart and sustainable building tech. would be easy to use.	3.80 (0.88)	0.69
PEOU3	Interacting with smart and sustainable building tech. would not require a lot of mental effort.	3.62 (0.97)	0.67
PEOU4	It would be easy to get smart & sustainable build. tech. to do what I/people want them to do.	3.55 (0.98)	0.65
	*Perceived usefulness*		
PU1	Smart and sustainable building tech. would improve my and other people’s performance.	3.71 (0.94)	0.69
PU2	Smart and sustainable building tech. would increase my and other people’s productivity.	3.66 (0.91)	0.86
PU3	Smart and sustainable building tech. would enhance my and other people’s effectiveness.	3.74 (0.90)	0.82
	*Behavioral intention to use smart building technology*		
BI1	I intend to use smart and sustainable building tech. in the near future (say 1 year).	3.29 (1.01)	0.65
BI2	I predict I would adopt smart and sustainable building tech. in the near future.	3.45 (0.97)	0.77
BI3	I plan to use smart and sustainable building tech. in the near future.	3.43 (0.98)	0.82

To ensure content validity, the draft questionnaire was reviewed by two academics and two building professionals and was pilot-tested with 10 part-time Masters’ students who had worked in engineering and building companies. The academics and building professionals indicated that the items measured what they were supposed to measure, supporting content validity. The students who participated in the pilot study indicated that the items were clear and they could complete the questionnaire on their own in 10 to 15 minutes.

### Data collection procedure and analysis approach

Two groups of part-time Masters’ students who had studied engineering at a Hong Kong university were invited to participate in the survey. They were assured that their responses would be kept anonymous and confidential. In addition, they were encouraged to provide the contact information of their friends and colleagues who had been working in the building and construction industry. Thus, all respondents of the survey were building professionals. Their participation was on a voluntary basis, and they could withdraw from the survey at any time they desired. All respondents were informed that the questionnaire aimed to solicit their opinions on building features and received no treatment of any kind during the survey process. In total, 1500 hard copies of the questionnaire were distributed. This chain referral sampling is applicable to exploratory and confirmatory studies in a specific context [40]. Recently, it has been used to identify the performance of environmental practices in Hong Kong [41] and to study construction practitioners’ perceptions toward carbon accounting in Australia [42]. After a two-month period, 440 questionnaires were completed and returned. Reminders were sent to non-responders. After another month, 152 further questionnaires were completed and returned. An inspection of all completed questionnaires showed that 49 respondents (33 and 16 in the first and second waves, respectively) had an education level of high school or less and worked as junior staff for less than a year. Hence, these 49 sets of data were discarded (similar to To et al. [41]), resulting in 543 usable responses.

Data were input to an IBM SPSS 24.0 file. Non-response bias was assessed based on the suggestions from Armstrong and Overton [43]. One hundred and thirty-six out of 407 usable responses from the first wave were randomly selected to compare with all 136 usable responses collected in the second wave. Results of independent sample *t*-tests on the items in the first two sections showed that there were no significant differences in responses from the first and second waves, indicating that non-response bias was not a problem. An IBM SPSS 24.0 was used to obtain the statistical properties of the collected data and to perform reliability tests of constructs. An IBM SPSS AMOS 24.0 was used to evaluate the measurement and structural models. Five indices were chosen to test the model-data fit. They included the normed chi-square (χ^2^/*df*), Tucker-Lewis index (*TLI*), comparative fit index (*CFI*), standardized root mean square residual (*SRMR*), and root mean square error of approximation (*RMSEA*). The criteria for an acceptable model fit were: χ^2^/*df* ≤ 0.3; *TLI* and *CFI* ≥ 0.9; *SRMR* and *RMSEA* ≤ 0.08 [44].

## Results

Out of the 1,500 questionnaires distributed, 543 usable sets of data were obtained, yielding a usable response rate of 36.2 percent. Four hundred and six respondents were males and 137 were females. Three hundred and thirty-nine respondents were in the age group of 20–29, followed by 148 in the age group of 30–39. Three hundred and thirty-one respondents had a bachelor’s degree and 189 had a Masters’ degree. Two hundred and twenty-two respondents were junior engineers and designers, followed by 200 engineers and designers. Approximately half of the respondents had a working experience of 4 years or more. Table 3 shows the demographic characteristics of the respondents.

**Table 3:** Demographic characteristics of respondents (*N* = 543).

		Number	Percent
Gender:	Male	406	74.8
	Female	137	25.2
Age group:	20–29	339	62.4
	30–39	148	27.3
	40–49	35	6.4
	50 or above	21	3.9
Education:	Professional diploma	18	3.3
	Bachelor	331	61.0
	Masters	189	34.8
	Doctorate	5	0.9
Position/job title:	Junior engineer/designer	222	40.9
	Engineer/designer	200	36.8
	Senior engineer/designer	84	15.5
	Engineering manager/manager	30	5.5
	Engineering director/director	7	1.3
Experience in the firm:	< 1 year	77	14.2
	1 to < 2 years	80	14.7
	2 to < 4 years	129	23.8
	4 to < 8 years	136	25.0
	8 years or more	121	22.3

The mean ratings of the items in the first two sections were compared between male and female respondents, between different age groups, between bachelor’s holders and respondents with other qualifications, between respondents with different professional levels, and between respondents with different years of working experience. Independent sample *t*-tests and analysis of variance tests showed that there were no significant differences between groups in most items. The mean ratings and standard deviations of the 22 items in the second section from all respondents are shown in Table 2. One-sample *t*-tests were performed to determine whether the mean ratings were greater than the midpoint of the 5-point scale. The results showed that the mean ratings of all 22 items were significantly greater than the midpoint of 3.0 (*p*<0.001).

### Salient features of smart and sustainable buildings

Table 1 presents the mean importance ratings of smart and sustainable buildings features. Building professionals considered an intelligent security to be the most important feature, followed by an intelligent and responsive fresh air supply, and an intelligent and responsive thermal control. However, building professionals considered an intelligent system that monitors people movement to be the least important feature, followed by a responsive system for harvesting rainwater, and a responsive acoustic environment.

### Structural equation modeling and hypothesis testing

Before performing structural equation modeling, the construct reliability of each construct was assessed using Cronbach’s alpha. Results showed that the Cronbach’s alpha values ranged from 0.76 to 0.83 as shown in Table 4, all of which are higher than the threshold of 0.7 as suggested by Nunnally [45] and Hair et al. [44].

**Table 4:** Cronbach’s alpha values, CR, AVE, and the inter-construct correlations.

	Cronbach’s α values	CR	AVE	FC	SN	IM	JR	PEOU	PU	BI
FC	0.80	0.80	0.57	***0*.*76***						
SN	0.78	0.79	0.55	0.34	***0*.*74***					
IM	0.76	0.77	0.52	0.26	0.42	***0*.*72***				
JR	0.80	0.80	0.58	0.26	0.35	0.50	***0*.*76***			
PEOU	0.76	0.76	0.45	0.31	0.33	0.29	0.54	***0*.*67***		
PU	0.83	0.83	0.63	0.10	0.30	0.44	0.36	0.37	***0*.*79***	
BI	0.78	0.79	0.56	0.54	0.36	0.39	0.40	0.43	0.37	***0*.*75***

Note: The square root of AVE of each construct is presented in bold italic font and shown on the diagonal and correlations between constructs are shown on the off-diagonal.

A confirmatory factor analysis was performed using IBM SPSS AMOS 24.0 with maximum likelihood estimation. The seven-factor measurement model produced the following fit indices: χ^2^/*df* = 2.50 (χ^2^ = 470.8, *df* = 188, *p*< 0.001); *TLI* = 0.92; *CFI* = 0.94; *SRMR* = 0.042; *RMSEA* = 0.053, indicating that the measurement model fitted the collected data appropriately. The standardized factor loadings ranged from 0.65 to 0.86 as shown in Table 2, higher than the minimum criterion of 0.40 as suggested by Hair et al. [44]. The average variance extracted (AVE) values for the seven constructs were calculated. The calculated values were greater than 0.5, except for PEOU which had a value of 0.45, generally supporting convergent validity [46]. Besides, the square root of AVE for each construct was greater than the inter-construct correlation values, thus supporting discriminant validity [46]. The Cronbach’s alpha values, composite reliabilities (CR), AVE and the inter-construct correlations are given in Table 4.

A single-factor measurement model was also created. The resulting fit indices for this model were χ^2^/*df* = 12.03 (χ^2^ = 2513.8, *df* = 209, *p* < 0.001); *TLI* = 0.43; *CFI* = 0.48; *SRMR* = 0.100; *RMSEA* = 0.143. The change in the chi-square value between the seven- and the single-factor model was significant (Δχ^2^ = 2043.0, Δ*df* = 21, *p*<0.001), supporting the conclusion that the seven-factor model should be used.

**Fig 2:**
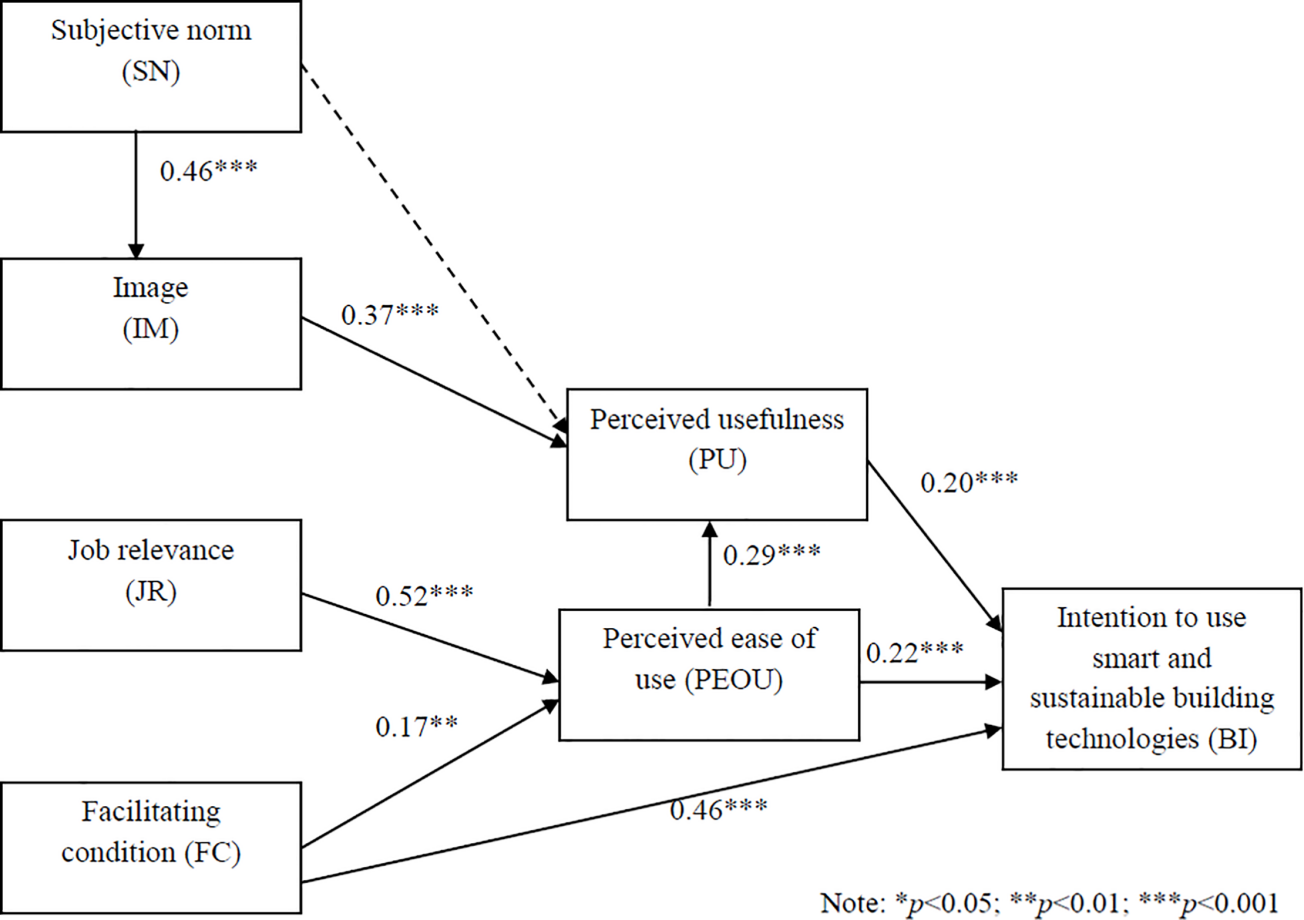
The final structural model.

The structural model was assessed using IBM SPSS AMOS 24.0 with maximum likelihood estimation. The seven-factor structural model shown in Fig 1 produced the following fit indices: χ^2^/*df* = 3.63 (χ^2^ = 717.9, *df* = 198, *p*< 0.001); *TLI* = 0.86; *CFI* = 0.88; *SRMR* = 0.093; *RMSEA* = 0.070, indicating that the hypothesized structural model did not fit the collected data well. After checking the modification indices and the standardized residuals, the model was refined iteratively to obtain the final structural model as shown in Fig 2. The fit indices of the final model were: χ^2^/*df* = 2.78 (χ^2^ = 550.1, *df* = 198, *p*< 0.001); *TLI* = 0.91; *CFI* = 0.92; *SRMR* = 0.061; *RMSEA* = 0.057, thus meeting the criteria as suggested by Hair et al. [44]. Fig 2 shows that the extended TAM was generally applicable to the prediction of building professionals’ intention to use smart and sustainable building technologies. FC was significantly related to PEOU and BI, supporting H4 and H5. SN was not significantly related to PU but it was significantly related to IM. Thus, H6 was not supported but H7 was. IM was significantly related to PU and JR was not related to PU. Hence, H8 was supported but H9 was not. PEOU was directly, significantly related to BI and indirectly, significantly related to BI through PU, thus supporting H2, H1, and H3.

Some new findings were obtained. The results of structural equation modeling showed that SN was not significantly related to PU (β = 0.07, *p* = 0.292, i.e., non-significant) and JR was significantly related to PEOU. These findings are unsurprising because the use of smart and sustainable building technologies would benefit building professionals’ during the design and construction phases of buildings (i.e., by having most of the smart and sustainable features that customers want in place) and building occupants during the operational phase of buildings. Hence, the feelings about JR are linked to PEOU rather than PU. After all, only part of PU can be realized in the design and construction phases of buildings. SN was found not to be significantly related to PEOU. This finding was consistent with the suggestion made by Yi, Jackson, Park, and Probst [47] who had explored information technology acceptance by individual professionals. Yi et al. [47] suggested that the identification effect could be taking place from SN to IM, and then from IM to PU, as what were found in the study. Furthermore, although many studies (for example, Davis et al. [17]; Gu et al. [16]; Venkatesh and Davis [15]) reported that PU was a stronger predictor of BI than PEOU. Our results showed the contrary. The standardized total effect of PEOU on BI was 0.28 (= 0.22+0.29×0.20) while the standardized total effect of PU on BI was 0.22. This was possibly because most prior studies had focused on the acceptance of computer and ICT technologies for personal use while our study investigated building professionals’ intention to use smart and sustainable building technologies for their design and engineering jobs as well as the betterment of building occupants, organizations, society and the environment. Hence, PEOU plays a more important role on which PU affects BI during the design and construction phase of buildings. Finally, our results showed that FC was the most significant predictor of BI. We calculated the total effect based on the direct and indirect effects. The standardized total effect of FC on BI was 0.51, much greater than the standardized total effect of JR on BI at 0.14, that of IM on BI at 0.07, and that of SN on BI at 0.03. This result was consistent with the findings of other researchers [41,48] who showed empirically that FC was directly linked to BI and actual behavior. Indeed, the resources and support from the organizations greatly and significantly influenced their employees to adopt smart and sustainable building technologies. Besides, building professionals’ perceptions of the relevance of smart and sustainable building technologies to their job also had an effect on their adoption of these technologies.

**Fig 3:**
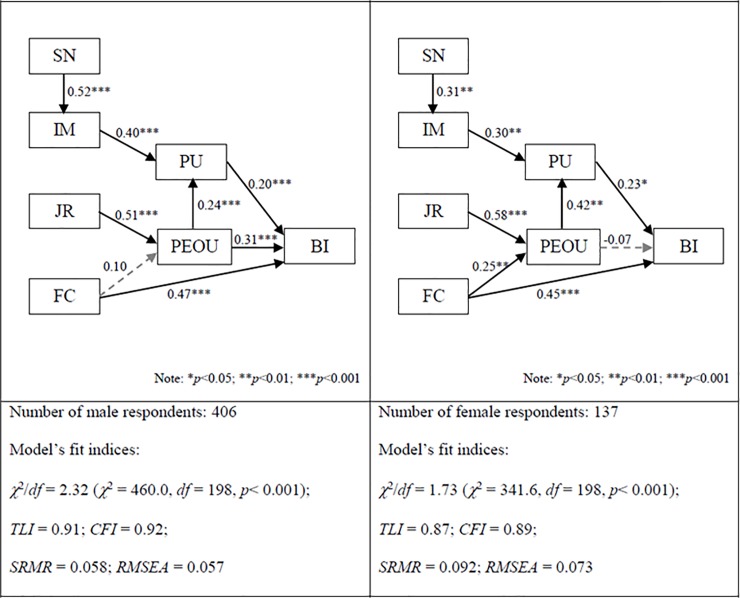
Effect of gender on the structural paths.

The comparison of the structural models for males and females revealed some differences in the structural paths. As shown in Fig 3, the path between FC and PEOU was found to be significant for females (β = 0.25, *p* < 0.01; sample size = 137) while this path was found to be not significant for males (β = 0.10, *p* > 0.05; sample size = 406), meaning that the resources and support from the organizations had greater effect for females than males on forming their perceptions of the ease of use of smart and sustainable building technologies. On the other hand, the paths between SN and IM and between IM and PU indicated that perceived usefulness was more strongly influenced by peer groups (i.e. SN) and respondents’ perceived status and image of using smart and sustainable building technologies (i.e. IM) for males than females. Fig 3 shows that the path between PEOU and BI was found to be not significant for females (β = -0.07, *p* > 0.05; sample size = 137) while this path was found to be very significant for males (β = 0.31, *p* < 0.001; sample size = 406), meaning that female respondents’ intention to use smart and sustainable building technologies was indirectly influenced by PEOU through PU.

**Fig 4:**
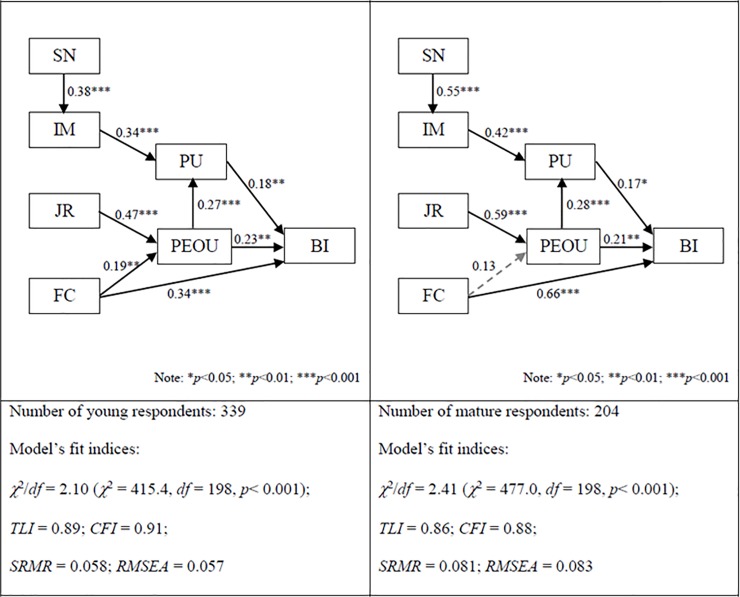
Effect of age on the structural paths.

The comparison of the structural models for young respondents aged 20–29 and mature respondents aged 30 or above revealed some differences in the structural paths. As shown in Fig 4, the path between FC and PEOU was found to be significant for young respondents (β = 0.19, *p* < 0.01; sample size = 339) while this path was found to be not significant for mature respondents (β = 0.13, *p* > 0.05; sample size = 204), meaning that the resources and support from the organizations had a greater effect for young respondents than mature respondents on forming their perceptions of the ease of use of smart and sustainable building technologies. On the other hand, the paths between SN and IM and between IM and PU indicated that perceived usefulness was more strongly influenced by peer groups (i.e. SN) and respondents’ perceived status and image of using smart and sustainable building technologies (i.e. IM) for mature respondents than young respondents.

## Conclusions

Smart and sustainable buildings are one of the building blocks of smart and green cities [4]. They can save energy, water, and material resources and provide adaptive and comfortable environment that enhances occupants’ well-being and productivity. Thus, the acceptance of smart and sustainable building technologies is of crucial importance. This study aimed at identifying Hong Kong building professionals’ views on smart and sustainable building features and how they perceived these technologies. Based on responses from 543 building professionals, an intelligent security system, an intelligent and responsive fresh air supply, and an intelligent and responsive thermal control were rated as the most important features of smart and sustainable buildings. However, building professionals indicated an intelligent system for monitoring people movement to be the least important. The most important and the least important features showed a security-privacy paradox. It imposes a challenge to building professionals on how to design a better building security and surveillance systems without inducing people’s feelings of being watched.

The results also showed that building professionals generally had a positive perception toward smart and sustainable building technologies, as the mean ratings of Image (IM), Job Relevance (JR), Perceived Ease of Use (PEOU), and Perceived Usefulness (PU) to be significantly greater than the midpoint of the 5-point Likert scale at 3.0. Building professionals also agreed that people who are important to them such as their seniors, colleagues, and professional friends think they should use smart and sustainable building technologies, as the meaning ratings of Subjective Norm (SN) items to be greater than 3.0. Besides, their organizations provide appropriate technical infrastructure to support them, as the meaning ratings of Facilitating Condition (FC) items to be greater than 3.0.

The results of structural equation modeling generally supported the view that the extended TAM could explain building professionals’ intention to use smart and sustainable building technologies. However, the final structural model showed that Behavioral Intention (BI) was directly, weakly, but significantly affected by Perceived Usefulness (PU) (β = 0.20, *p* < 0.001) and Perceived Ease of Use (PEOU) (β = 0.22, *p* < 0.001). PU was moderately significantly affected by Image (IM) (β = 0.37, *p* < 0.001) while Image (IM) was moderately significantly affected by Subjective Norm (SN) (β = 0.46, *p* < 0.001). PEOU was strongly significantly affected by Job Relevance (JR) (β = 0.52, *p* < 0.001) and weakly significantly affected by Facilitating Condition (FC) (β = 0.17, *p* < 0.001). Nevertheless, the strongest predictor of Behavioral Intention (BI) was Facilitating Condition (FC) (Direct effect: β = 0.46, *p* < 0.001). Building professionals’ intention to use smart and sustainable building technologies was strongly influenced by the supports they had from the organizations. Thus, strong commitment from top management to use smart and sustainable technologies is crucial. If organizations provide appropriate technical infrastructure including platforms, systems, software and hardware, training, and technical assistance to those professionals for the design and construction of smart and sustainable buildings, building professionals will have a much higher intention to use such technologies. Moreover, Job Relevance (JR) also influenced Behavioral Intention (BI) indirectly through Perceived Ease of Use (PEOU). Hence, smart and sustainable building technology providers, building owners, and executives of building consulting and contracting companies should try their best to provide resources such as training and software and to stress the importance and relevance of smart and sustainable building technologies for smart and green buildings and cities. They can organize seminars, forums, exhibitions, and conferences to promote and update building professionals about the development and application of smart and sustainable building technologies. These practices can improve building professional’s perceptions of Facilitating Condition (FC) as well as job relevance (JR).

Finally, some differences were observed between female and male respondents and between young and mature respondents. It was found that Facilitating Condition (FC) had a significant effect on Perceived Ease of Use (PEOU) for females but not for males or for young respondents but not for mature respondents. Besides, Perceived Usefulness (PU) was more strongly influenced by Image (IM) and indirectly by Subjective Norm (SN) for males than females or for mature respondents than young respondents.

## Supporting information

S1 File Data file in IBM SPSS 24.0 format. (SAV)
